# CRISPR/Cas9 cleavage of viral DNA efficiently suppresses hepatitis B virus

**DOI:** 10.1038/srep10833

**Published:** 2015-06-02

**Authors:** Vyas Ramanan, Amir Shlomai, David B.T. Cox, Robert E. Schwartz, Eleftherios Michailidis, Ankit Bhatta, David A. Scott, Feng Zhang, Charles M. Rice, Sangeeta N. Bhatia

**Affiliations:** 1 Department of Health Sciences and Technology, Massachusetts Institute of Technology, Cambridge, MA 02139, USA; 2 Laboratory of Virology and Infectious Disease, Center for the Study of Hepatitis C, The Rockefeller University, New York, NY 10065, USA; 3 Department of Medicine, Brigham and Women’s Hospital, Boston, MA 02115, USA; 4 Division of Gastroenterology and Hepatology, Weill Cornell Medical College, New York, NY 10065, USA; 5 Department of Electrical Engineering and Computer Science, Massachusetts Institute of Technology, Cambridge, MA 02139, USA; 6 Broad Institute, Cambridge, MA 02139, USA; 7 Howard Hughes Medical Institute, Cambridge, MA 02139, USA; 8 Koch Institute for Integrative Cancer Research, Massachusetts Institute of Technology, Cambridge, MA 02139, USA; 9 Department of Biology, Massachusetts Institute of Technology, Cambridge, MA 02139; 10 Department of Brain and Cognitive Sciences, Massachusetts Institute of Technology, Cambridge, MA 02139; 11 McGovern Institute for Brain Research, Massachusetts Institute of Technology, Cambridge, MA 02139

## Abstract

Chronic hepatitis B virus (HBV) infection is prevalent, deadly, and seldom cured due to the persistence of viral episomal DNA (cccDNA) in infected cells. Newly developed genome engineering tools may offer the ability to directly cleave viral DNA, thereby promoting viral clearance. Here, we show that the CRISPR/Cas9 system can specifically target and cleave conserved regions in the HBV genome, resulting in robust suppression of viral gene expression and replication. Upon sustained expression of Cas9 and appropriately chosen guide RNAs, we demonstrate cleavage of cccDNA by Cas9 and a dramatic reduction in both cccDNA and other parameters of viral gene expression and replication. Thus, we show that directly targeting viral episomal DNA is a novel therapeutic approach to control the virus and possibly cure patients.

Hepatitis B virus (HBV) chronically infects over 250 million people worldwide. Chronically infected individuals are at an increased risk for deadly complications, including cirrhosis, end-stage liver disease and hepatocellular carcinoma, resulting in approximately 600,000 deaths per year^1^. HBV is a member of the *Hepadnaviridae* family and its life cycle involves both DNA and RNA intermediates. The HBV genome exists in the nuclei of infected hepatocytes as a 3.2kb double-stranded episomal DNA species called covalently closed circular DNA (cccDNA). cccDNA is a key component in the HBV life cycle, since it is the template for all viral genomic and subgenomic transcripts^2^. Currently approved HBV therapies act post-transcriptionally to inhibit viral replication and thus fail to target or eliminate the cccDNA pool, which exhibits extraordinary stability and persistence^3^. Consequently, these drugs must often be taken indefinitely to prevent viral rebound. Agents that act directly on viral DNA to deplete this reservoir may represent more desirable and possibly curative therapeutic alternatives^4^.

To this end, targeted nucleases may provide an efficient and specific way to damage the HBV genome while sparing host genomic DNA^5^,^6^,^7^. Targeted nucleases catalyze double-stranded DNA break (DSB) formation, which leads to the formation of mutagenic insertions and deletions (indels) through error-prone nonhomologous end-joining (NHEJ) at the target DNA locus. Recently, the type II CRISPR-Cas system of *Streptococcus pyogenes* SF370 has been adapted as an RNA-guided, sequence-specific DNA nuclease for use in mammalian cells^8^,^9^. CRISPR/Cas9 and other genome engineering technologies have been employed to design candidate therapeutics via gene targeting, knockout of beneficial host genes, and mutation of integrated viruses^10^, and we sought to further study the application of CRISPR/Cas9 to direct targeting and cleavage of HBV cccDNA. We hypothesized that by directly targeting the HBV genome for cleavage using CRISPR/Cas9, we could suppress HBV by mutagenizing critical genomic elements or decreasing the stability of cccDNA and other viral intermediates through repeated linearization of the circular genomes (Fig. 1a).

## Results

### CRISPR/Cas9 design and validation

**Figure 1:**
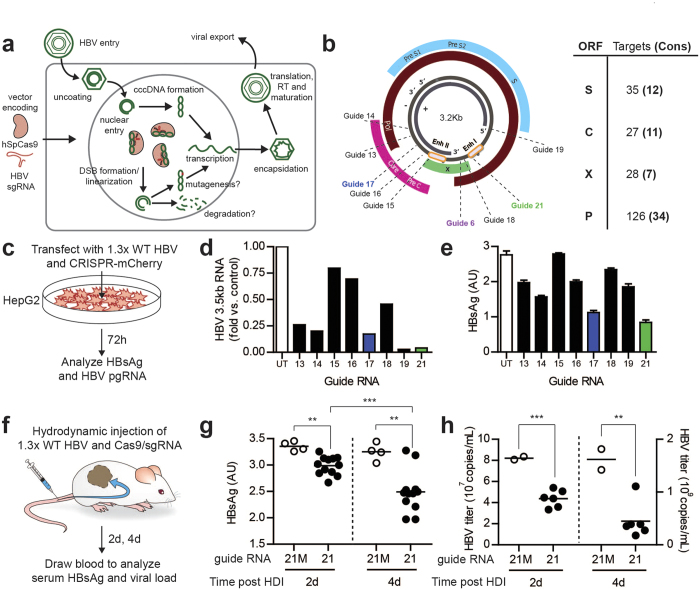
Transiently transfected CRISPR constructs exhibit anti-HBV activity. (**a**) Schematic of HBV life cycle and putative anti-HBV effect of CRISPR constructs; Cas9-mediated DSB formation should linearize the small, episomal cccDNA repeatedly, potentially leading to indel formation (generating less-fit viral mutants) or even degradation. (**b**) (left) HBV genome organization and location of target sequences for several tested guide RNA constructs. (right) Table of all possible CRISPR target sites in each HBV ORF, including number of possible target sites in conserved genomic regions. (**c**) Experimental schematic for (**d**-**e**): HepG2 cells are co-transfected with 1.3x WT HBV and sgRNA/Cas9-2A-mCherry construct, and (**d**) intracellular HBV pregenomic RNA and (**e**) secreted HBsAg are quantified after 72 hours. Data shown were generated in one representative experiment, with intracellular pgRNA harvested from one pellet and HBsAg collected from replicate wells per group; all data are consistent across three independent experiments. (**f**) Experimental schematic for (**g**-**h**): 1.3x WT HBV and sgRNA/Cas9-2A-mCherry are delivered to the livers of immunodeficient NRG mice via hydrodynamic injection, and (**g**) HBsAg and (**h**) secreted HBV titer are quantified in mouse blood at 2 and 4 days post injection. 21M: guide RNA with 5 bp mismatch from g21. Data shown are from one representative experiment, and consistent across multiple experiments. UT: ‘untargeted’ guide RNA (no target sequence in HBV genome). *p < 0.05 for selected comparison; ^**^p < 0.01 for selected comparison; ***p < 0.001 for selected comparison as assessed by two-tailed t-test.

Using the CRISPR online design tool ( http://www.genome-engineering.org/crispr/), we generated 24 single guide RNAs (sgRNAs) targeting the HBV genome (Fig. 1b, Table S1). Target sequences were chosen in order to maximize conservation across viral genotypes (Fig S1) and minimize homology to the human genome. Based on these criteria, we only designed guides targeting the core, polymerase and X ORFs, but numerous Cas9 target sites also exist in the S ORF (Fig. 1b). To evaluate the efficacy of selected sgRNAs (Fig. 1b) in targeting HBV, we co-transfected the HepG2 hepatoma cell line with an HBV-expressing plasmid and constructs expressing Cas9 and individual gRNAs, and measured the production of HBV 3.5kb RNA (encoding pre-genomic RNA (pgRNA), the template for reverse transcription) as well as the secretion of HBV surface antigen (HBsAg) into the medium, two reliable indicators for viral gene expression and replication (Fig. 1c). sgRNAs 17 and 21 (sg17 and sg21) consistently led to a decrease in pgRNA levels and HBsAg production (Fig. 1d,e). While other sgRNAs (sg14 and sg19) generated similar decreases in HBV pgRNA, these guides did not have as large an effect on HBsAg secretion as did sg17 and sg21. This source of this discrepancy is not entirely clear, but may be related to targeting different locations along the HBV genome that exert effects on pgRNA transcription but do not suppress HBsAg expression.

Given their strong effect on both viral parameters measured, we proceeded with sg17 and sg21, as well as sg6 - identified from previous pilot experiments. In addition, to investigate the effect of multiplex targeting of HBV DNA in order to impact multiple viral elements, we co-transfected HepG2 cells with control sgRNA, sg17, sg21, or a combination of sg17/sg21. The combination of two guide RNAs targeting HBV led to stronger reductions in HBsAg and HBV 3.5kb RNA as compared to the single guide RNAs (Fig S2).

### Confirmation of anti-HBV effect *in vivo*

We next sought to evaluate the antiviral effect of Cas9 *in vivo,* to ensure that our anti-HBV constructs functioned appropriately in primary hepatocytes. To do this, we used a mouse model of HBV, where HBV and Cas9/sgRNA plasmids were introduced to the liver of immunodeficient mice (NRG) by hydrodynamic injection (HDI)^11^ (Fig. 1f). In the case of proof-of-concept studies such as this, we endeavor to minimize the use of animal subjects. Thus, the complete battery of *in vivo* experiments described below were performed with only sg21 and its mutated control, although similar results were replicated with other guides (data not shown). Animals expressing Cas9 and sg21 in this model showed a progressive suppression of HBV expression as compared to controls expressing Cas9 and a mutated sgRNA (sg21M; 3’ 5 bp mismatch), reflected by a decrease in HBsAg secretion and a 4-fold decrease in viremia at day 4 post injection (Fig. 1g,h).

### Sustained Cas9/sgRNA expression dramatically inhibits HBV

Recent genome-wide CRISPR knockout studies have shown that sustained Cas9/sgRNA expression induces progressively greater indel formation over time in mammalian cells^12^. Based on this information and encouraged by our initial results, we evaluated the efficacy of sustained Cas9/sgRNA expression in inhibiting HBV using a model that more reliably recapitulates HBV life cycle components. For these studies, we used the HepG2.2.15 hepatoblastoma cell line, which harbors both a functional HBV integrated form and cccDNA, and constitutively produces infectious virions^13^ (Fig S3). Because cccDNA cannot be reliably quantified or detected in plasmid co-transfection or HDI systems, the HepG2.2.15 system is more ideal for investigating CRISPR/Cas9-mediated clearance of this viral species.

**Figure 2:**
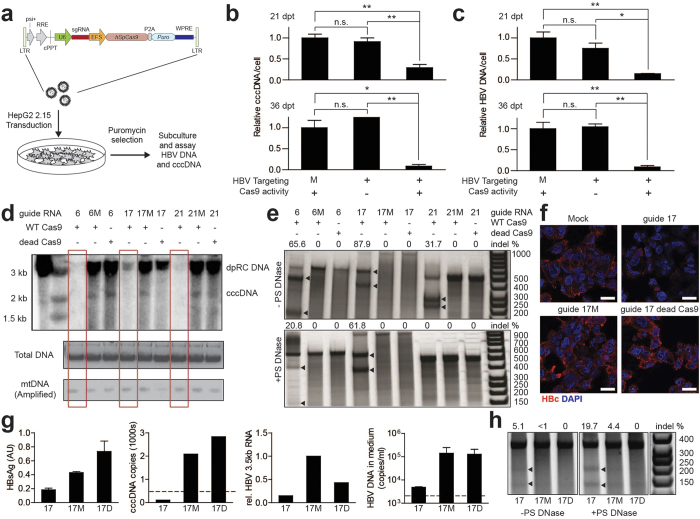
Sustained expression of CRISPR machinery enables large reductions in HBV DNA and cccDNA. (**a**) Schematic of lentiviral vector and experimental strategy for sustained CRISPR expression. (**b**-**c**) CRISPR constructs targeting HBV cause progressive reduction in (**b**) cccDNA and (**c**) total HBV DNA levels dependent on successful targeting of viral DNA and Cas9 nuclease activity; data shown are from one representative experiment pooled across 3 separate HBV-targeting guides (sg6, sg17, sg21), and consistent across multiple independent transduction experiments. (**d**) Southern blot of HBV DNA forms using Hirt’s extraction (to deplete high-molecular weight DNA), shows HBV-targeted sgRNAs with nuclease-active Cas9 generate near-total reduction in cccDNA. (**e**) Surveyor assay to detect indel formation in total HBV DNA (top) and episomal HBV DNA, enriched by treatment with plasmid-safe DNase (bottom); lentiviral transduction enables high levels of cutting of HBV. Arrowheads depict surveyor digestion products. Expected PCR product sizes for sg6, sg17 and sg21 are respectively 599, 946 and 507 bp. Approximate sizes of surveyor digestion products for sg6, sg17 and sg21 are respectively: 429 + 170, 570 + 376, 275 + 232. (**f**) Immunofluorescent imaging of HBV Core protein demonstrates large reduction in Core staining upon targeting by sg17 specifically against the Core ORF. (**g**-**h**) Cas9/gRNA-transduced Hep-NTCP cells are cocultured with HepG2.2.15 cells to infect them with HBV followed by depletion of HepG2.2.15 cells using puromycin selection (Schematic in Fig S6 left). (**g**) From left to right, HBsAg secretion, cccDNA copies, levels of HBV 3.5kb RNA relative to 5 bp mismatch control, and titer of HBV DNA in culture medium show that Cas9/sg17 reduce HBV infection in *de novo* infection. 17M: 5 bp mismatch control. 17D: dead Cas9 with g17. Data shown are from one representative experiment, and consistent across experiments. (**h**) Surveyor assay performed on DNA untreated (left) or treated (right) with Plasmid-Safe DNase to remove non-episomal viral forms. Arrowheads indicate indel formation. (**b**-**c**) ^*^p < 0.05 for selected comparison; ^**^p < 0.01 for selected comparison, as assessed by one-way ANOVA with Tukey post-hoc test.

We transduced HepG2.2.15 cells with Cas9-2A-Puro lentiviruses encoding Cas9 and individual sgRNAs (sg6, sg17, sg21) chosen based on our initial results, and treated cells with puromycin to select for transduced cells (Fig. 2a). As controls, cells were also transduced with constructs containing sgRNAs and a nuclease deficient Cas9 (D10A/H840A; dCas9) to control for nuclease-independent effects of Cas9 on viral fitness, or WT Cas9 with mutated sgRNAs (gXM) to control for guide sequence-independent effects. Cas9/sgRNAs induced robust suppression of HBV DNA release (77-95% decrease across different sgRNAs), HBeAg secretion, and viral mRNA production (greater than 50%) (Fig S4). We next analyzed the effect of Cas9-mediated cleavage on the abundance of non-integrated viral forms, composed mainly of cccDNA (See Methods). Quantitative PCR showed a robust reduction in total HBV DNA and in cccDNA. Pooling the data from sg6, sg17, and sg21, cccDNA reduction progressed from 71 + /−7% reduction at day 21 to 92 + /−4% at day 36 post transduction (Fig. 2b,c, data for individual sgRNAs in Fig S5). These results were confirmed by directly analyzing low molecular weight DNA from transduced cells by Southern blot (Fig. 2d). cccDNA and its deproteinated relaxed circular form (dpRC DNA) precursor were greatly depleted in Cas9/sgRNA transduced cells. In contrast, when total HBV DNA was analyzed, no substantial reduction in the levels of integrated HBV DNA was detected (Fig S6).

We then performed the Surveyor assay on HBV, to directly determine whether the viral DNA was cleaved and repaired via error-prone NHEJ similar to genomic targets of CRISPR/Cas9. Interestingly, analysis of total HBV DNA forms for indel formation, an indirect measure of Cas9-mediated cleavage, revealed a substantial mutagenesis rate (Fig. 2e top). When we performed the same analysis after depleting integrated genomic HBV, we observed a lower rate of indel formation (0% vs 32%, 62% vs 88% and 21% vs 66% for guides sg21, sg17 and sg6, respectively) (Fig. 2e bottom). Notably, however, this analysis method cannot detect Cas9-mediated cleavage of cccDNA followed by exonuclease-mediated degradation from the newly-formed free DNA ends (instead of re-ligation by NHEJ), and may be limited by the very small amount of episomal HBV remaining at late time points (Fig. 2d, Fig S4). Consistent with high levels of indel formation in the core ORF targeted by sg17, immunostaining for HBV core protein (HBc) revealed a robust reduction in HBc levels in sg17-expressing cells as compared to controls (Fig. 2f). Because long-term expression of Cas9 and guide RNAs can lead to off-target cleavage at sites with homology to the target sequence, we then performed next-generation sequencing at several computationally predicted off-target sites for sg6, sg17, and sg21. Within the sensitivity of our assay (< 0.3% based on read depth), we detected no indel formation at the 8 off-target sites that we surveyed after constitutive expression of Cas9 and sgRNAs for over four weeks (Fig. S7a). This observed specificity may be due to the large sequence differences between viral and human genomic DNA (Fig. S7b-d). These encouraging results still did not exclude the possibility that some of the antiviral effects of Cas9 in the HepG2.2.15 system occur through mutations in integrated HBV DNA, thereby reducing the fitness and/or persistence of virions produced from mutated loci rather than acting directly on episomal DNA. Since integration of HBV DNA into the host human genome is not part of the canonical HBV life-cycle, we next evaluated the effects of Cas9 targeting in the context of *de novo* HBV infection, where episomal cccDNA serves as the only template for viral gene expression and replication.

### Cas9 cleaves cccDNA and inhibits de novo HBV infection

To evaluate our anti-HBV CRISPR/Cas9 strategy in a setting of *de novo* infection, we used HepG2 cells overexpressing the HBV receptor NTCP (Hep-NTCP)^14^, which are permissive to infection with HBV. Because sg17 showed the highest levels of cccDNA mutagenesis in our HepG2.2.15 experiments, these cells were transduced with Cas9/sg17, Cas9/sg17M, or dCas9/sg17 lentiviruses, co-cultured with HBV producing HepG2.2.15 cells, and selected with puromycin to get rid of non-transduced Hep-NTCP and co-cultured HepG2.2.15 cells (Fig S8 left). Alternatively, Hep-NTCP cells were selected with puromycin following transduction and subsequently infected with HBV-positive patient serum (Fig S8 right). When the transduced Hep-NTCP were infected with cell culture-produced virus, Cas9/sg17 greatly abrogated productive HBV infection, as reflected by reduction in HBsAg and HBV DNA secretion, as well as 3.5kb RNA and cccDNA levels, compared to controls (Fig. 2g); this was confirmed by infection with patient-derived virus (Fig S9). While nuclease-deficient Cas9 also reduced viral 3.5kb RNA abundance in this system, this finding fits with other reports that dCas9 binding can inhibit transcription in mammalian cells^15^. Surveyor assay performed using DNA from cells infected *de novo* with HepG2.2.15-derived virus confirmed direct Cas9-mediated mutagenesis of HBV episomal DNA (Fig. 2h). Although some mutagenesis was also detected when the mutated sg17M was used, this most likely was due to low-level cleavage with DNA bulge-containing guide RNAs^16^. This finding provides direct evidence that Cas9 is capable of targeting episomal forms of the virus, and exerting anti-HBV effects by directly targeting cccDNA.

## Discussion

Although largely unexplored in mammalian systems, bacteria and archaea utilize sequence specific DNA nucleases to interfere with viral replication^17^. Inspired by CRISPR’s evolutionary origins, we aimed to exploit the antiviral activity of Cas9 to target HBV DNA in mammalian cells. We show that targeting multiple conserved regions of HBV with Cas9 results in robust suppression of viral replication and direct mutagenesis and depletion of cccDNA. While integrated forms of HBV DNA were not depleted by Cas9 cleavage, these forms should not contribute to viral rebound *in vivo*^18^, and Cas9-driven mutagenesis of these sequences nonetheless would damage the viability of viral proteins generated from integrants. The unique advantages of the CRISPR/Cas9 system (such as multiplexed targeting) are of interest in developing antiviral applications, and indeed, very recently other groups have published examples of Cas9 cleavage of HBV in multiple model systems^19^,^20^,^21^,^22^. Our work provides an extension beyond these complementary studies, by demonstrating the anti-HBV effects of sgRNAs *specifically* targeting highly conserved regions of HBV *in vitro* and *in vivo*, by directly confirming mutagenesis in cccDNA in a *de novo* infection model of HBV, and extending this antiviral activity to patient-derived virus. Additionally, our finding that appropriately chosen virus-targeting sgRNAs can avoid inducing off-target cleavage, even upon sustained Cas9/sgRNA expression, strengthens the case for selecting viral targets as good candidates for CRISPR/Cas9 therapeutic use^23^.

Interestingly, while Cas9/sg17 was efficient in suppressing infection and in directly cleaving nuclear cccDNA, Cas9/sg21 efficiently cleaved only integrated but not episomal DNA, which resulted in a lack of activity for Cas9/sg21 in *de novo* infection experiments (data not shown). The reason for this is unclear and warrants further study. Cas9 is a large multi-domain protein, and thus one hypothesis is that particular regions of the HBV genome are differentially accessible to Cas9 because of the tightly packed physical architecture of cccDNA. This underscores the importance of using models of authentic cccDNA to investigate therapeutic applications of targeted nucleases for HBV, and suggests that a careful selection of targets and guides will be required to achieve a substantial mutagenesis and depletion of viral DNA. In addition, our proof of concept experiments show that multiplexing sgRNAs can generate stronger antiviral effects (Fig S2), suggesting that this strategy may further maximize CRISPR-mediated restriction of components of the viral life cycle, possibly including cccDNA stability.

This study provides a proof of concept, but clinical translation of CRISPR/Cas9 systems to cure HBV will require some advances over the work described here. First, an exhaustive profiling of possible Cas9 target sites on cccDNA can uncover optimal target sites based on cccDNA accessibility and sgRNA binding properties. Secondly, delivery of Cas9/sgRNA constructs *in vivo* will require the use of clinically relevant delivery vectors such as AAV, which may require additional modifications such as switching to smaller Cas9 orthologs to save packaging size^30^. Finally, although we could not find evidence of off-target cutting in our directed sequencing, possibly due to the low homology between viral and human genomic Cas9 targets, an extensive genome-wide profiling of off-target effects is warranted.

The unusual persistence of cccDNA is currently the major obstacle for curing chronic HBV infection. To eliminate the virus and to prevent possible re-activation, it is probably necessary to eliminate all or at least the vast majority of episomal DNA from hepatocytes through a combination of exogenous treatment (presented here) and immune-mediated endogenous clearance. CRISPR/Cas9-mediated therapy may synergize with currently-used RT inhibitors, which should block the formation of new molecules of cccDNA via re-entry of newly synthesized replicative forms to the nucleus. The developments proposed above represent an active area of investigation for groups looking for ways to use CRISPR in a therapeutic fashion more broadly, which may accelerate progress toward an anti-HBV CRISPR therapeutic.

In summary, these results constitute the first example of CRISPR/Cas9 systems directly targeting an authentic pathogenic virus with episomal DNA, and demonstrate the potential for cccDNA-directed antiviral therapy using Cas9, which may represent a significant step towards the cure of chronic HBV infection. The results demonstrated here may also be used to inform the development of CRISPR/Cas9-based therapeutics for other DNA viruses, such as herpesviruses and papillomaviruses that use an episomal DNA as a template for their gene expression and replication.

## Methods

### Tissue culture and transfection experiments

HepG2 or HepG2.2.15 cells were maintained in DMEM and 10% fetal calf serum (FCS) as previously described^24^. For transfection experiments, the 1.3xHBV plasmid was used as previously described^25^ . Briefly, an over-length HBV genome (*adw* strain) of 4195bp was produced, harboring a 5’ terminus of the unique EcoRV site (nt 1043, considering EcoRI unique site in the original 3.2kb HBV construct as nt number 1) and a 3’ terminus of the unique Taq1 site (nt 2017). This EcoRV-TaqI fragment was inserted between the SmaI-AccI unique sites of a pGEM-3Z plasmid, respectively. This plasmid expresses all HBV gene products and generates infectious virions secreted to the medium. Transfection was carried out using the TransIT- 2020 transfection reagent (MIRUS) according to the manufacturer’s instructions.

### Total HBV and cccDNA extraction and analysis

Cell pellets or mediums were collected and DNA was extracted using the QIAamp DNA blood mini kit (QIAGEN, cat No 51104) or QIAamp Minielute Virus spin kit (QIAGEN, cat No 51104), respectively. DNA was extracted according to the manufacturer’s protocol, and final product was eluted in 60ul of water. 5ul was taken for a QPCR. PCR for total HBV DNA using the TaqMan® Universal PCR Master Mix (Applied Bio systems, Cat No 4304437) and the following primers and probe: 5’CCGTCTGTGCCTTCTCATCTG3’ (sense), 5’AGTCCAAGAGTCCTCTTATGTAAGACCTT3’ (anti sense), 5- /56-FAM/CCG TGT GCA /ZEN/CTT CGCTTC ACCTCT GC/3IABkFQ/ -3 (probe). PCR was done using the Roche LightCycler **®**480 PCR machine. Quantification was done according to a standard curve composed from 2xHBV plasmid in a concentration range of 10^9^–10^1^ copies.

For cccDNA extraction and analysis, DNA extracted from cells was subjected to ON digestion with a plasmid-safe DNase (Epicentre) as previously described^14^. Following enzyme inactivation at 70**°** C for 30min, DNA was subjected to real-time PCR using SYBR**®** Premix Ex Taq (TaKaRa) following a previously described protocol^14^ and using cccDNA specific primers previously described by Glebe *et al.*^26^. The primers used for cccDNA amplification: 5’TGCACTTCGCTTCACCTF3’ (sense) 5’ AGGGGCATTTGGTGGTC3’ (anti sense). For quantification, a standard curve derived from decreasing concentrations of 2xHBV plasmid was used. PCR was performed using the Roche LightCycler **®**480 PCR machine.

### Hirt’s extraction

Hirt’s extraction was performed as previously described^27^. About 60% of final DNA extract derived from one well of a 6-well plate was run for Southern blot analysis.

### Southern blot analysis

Total DNA or Hirt’s extract were run on 0.8% agarose-TAE gel, followed by denaturation and southern blotting to a Hybond N nylon membrane (Amersham). Viral DNA was detected by hybridization with a ^32^P random primed HBV probe, using the Prime-It II Random Primer Labeling Kit (Agilent Technologies, Cat No 300385). Following incubation and washing, membrane was visualized by phosphorImager and later exposed to film.

### HBV mRNA analysis

Total RNA was isolated via TRIZOL RNA/DNA extraction. After being subjected to DNaseI treatment, RNA was quantified using a NanoDrop and first-strand cDNA was synthesized using SuperScript® III RT kit (INVITROGEN). Quantitative PCR for 3.5kbRNA or total HBV RNA was carried out with SYBR Green PCR master Mix (Applied Biosystems) and using specific primers previously described^14^. In each reaction an RT negative control was included to rule-out DNA carry over.

### Immunostaining for HBV Core antigen

Cells were grown on chambered coverglasses (Lab-Tek, Rochester, NY), washed with PBS, and then fixed with 4% paraformaldehyde. Cells were washed again (3x PBS) and treated with 100 mM glycine solution in PBS. After permeabilization with 0.1% Triton X-100 in PBS and treated with Image-iT™ FX signal enhancer (Life Technologies). Cells were blocked in PBS/10% goat serum (Jackson Immunosearch)/1% BSA. HBV core staining was achieved by using a polyclonal rabbit anti-HBV core antibody (Dako, CA) diluted 1:1000 in PBS/0.1% BSA (18 h at 4 ^o^C). As a secondary antibody a goat-anti-rabbit labeled with AlexaFluor594 (Life Technologies) diluted 1:2000 in PBS/0.1% BSA was used. Nuclear staining was achieved using DAPI treatment. Image acquisition was performed in a Zeiss confocal microscope and image analysis was done using ImageJ (NIH, Bethesda, MD).

### Hepatitis B e Antigen and HBsAg ELISA

The HBV e Antigen ELISA was performed using the Hepatitis B e Antigen (HBeAg) chemiluminescence Immunoassay kit (Autobio Diagnostics Co, Cat No.CL0312-2) according to the manufacturer’s instructions. For HBsAg detection, 100ul medium was loaded on ELISA plates coated with mouse monoclonal anti HBsAg antibodies (Bio-Rad, GS HBsAg EIA 3.0, Cat. No. 32591). ELISA was carried out according to the manufacturer’s instructions. Plates were read using the FLUOstar Omega luminometer (BMG LABTECH). HBsAg positivity (cutoff) was calculated as an average of 3 negative controls+0.07.

### Animal studies

NRG mice were injected with a mixture of 15ug 1.3xHBV plasmid, 20ug the CRISPR expressing plasmid and 10ug of luciferase expressing plasmid (to control for expression efficiency) using the hydrodynamic delivery (HDD) technique, as previously described^11^. Plasmids were dissolved in PBS in a volume corresponding to 0.09 times the animal weight (in grams) and the mixture was injected through the tail vein in 7-9 sec. To verify successful injection and gene expression, animals were visualized by the IVIS machine at various time points after HDD. Mice were housed in an AAALAC-accredited facility and all experiments were performed in accordance with the Guide for the Care and Use of Laboratory Animals. All procedures outlined in the study were approved by The Rockefeller University’s Institutional Animal Care and Use Committee (IACUC).

### Lentivirus production

293T cells were co-transfected with the sgRNA-Cas9-2A-Puro lentiviral vectors (Fig. 2A) and a 2nd-generation lentiviral packaging system (psPAX2 and pMD2.G) at a ratio of 3:2:1. Cells were washed 24h after transfection, supernatant was collected every 24h from 48-96h post transfection, and cell debris was removed by centrifugation. Lentivirus was concentrated by ultracentifugation for 1.5h at 16,600x g, incubated O/N in Optimem at 4C, then resuspended in Optimem, aliquoted and frozen at -80C the next day, prior to use.

### Transduction and drug treatment experiments

HepG2.2.15 cells were maintained as noted above until transduction, and then transduced with sgRNA-Cas9-2A-Puro lentiviruses at a confluence of 50-60% with an MOI of 1. Transduction was performed by mixing lentivirus aliquots with standard HepG2.2.15 culture medium, washing cells and adding lentivirus-containing medium at 2.5 mL/well in a 6-well plate, centrifuging for 1h at 200 x g and then incubating for an additional 23h. 24h after addition of lentivirus, cells were washed 3x and incubated in standard medium+2.5 ug/mL puromycin to remove untransduced cells. Puromycin selection was continued for 48h, then cells were washed 3x and maintained in standard medium. Transduced cells were then continually passaged upon reaching 80% confluence; at each passage, cells were counted, cell pellets were harvested for each condition, and a portion of the remaining cells were reseeded at 10% confluence.

### Cloning of CRISPR Constructs

Cas9 constructs with guide RNAs targeting sequences present in the HBV genome integrated into the HepG2.2.15 cell line were used for the described experiments. Guide RNAs were of the form 5’-G(N19)-3’ with their target sequences having the form of 5’-G(N19)-NGG-3’. Oligos to create gRNAs were cloned into the lentiCRISPR construct described in (12) or PX330a described in (21).Two sets of control constructs were generate: Mismatched guide RNA control constructs for promising guide RNA molecules were created by ligating in oligos to PX330a or lentiCRISPR that contained 5 basepair mismatches at the 3’ end of the spacer, but were otherwise identical to constructs designed to target HBV. Cas9 D10A/H840A nuclease dead control constructs were generated by digesting lentiCRISPR plasmid guide RNA containing constructs with *BamHI* and *XbaI* (ThermoScientific) and then inserting a PCR amplified D10A/H840A Cas9 using Gibson Assembly. D10A and H840A are mutations that are sufficient to abolish the nuclease activity of *S. pyogenes* SF370 Cas9^8^,^28^.

### Surveyor

Targeted loci were amplified by PCR using Phusion Flash (NEB) or Heruclase II (Agilent) polymerases and primers listed in Table S2. For sg17 and sg21, two separate sets of primers were designed for each guide in order to optimize the PCR reaction; sets F2 and R2 are recommended. PCR products were gel or PCR-purified using Qiagen kits and subject to the Surveyor assay (Transgenomics) according to the manufacturer’s instructions. Indel rate for surveyor was calculated as described in reference (8).

### Deep sequencing for on-target and off-target cleavage

Potential off target sites were identified using the CRISPR online design tool (crispr.mit.edu). 8 of the top chromosomal off-target sites for guides sg6, sg17 and sg21, along with on-target sites, were PCR amplified with primers designed to attached Illumina P5 adapters and sample-specific barcodes. PCR products were purified using QIAQuick PCR Spin Columns (QIAGEN), quantified with a Qubit 2.0 Fluorometer (Life Technologies) and pooled in an equimolar ratio. Amplicons were then sequenced with the Illumina Miseq Personal Sequencer. Indel frequencies for NGS reads were calculated in a manner similar to Hsu *et al*.^29^ in Geneious.

## Additional Information

**How to cite this article**: Ramanan, V. *et al.* CRISPR/Cas9 cleavage of viral DNA efficiently suppresses hepatitis B virus. *Sci. Rep.* **5**, 10833; doi: 10.1038/srep10833 (2015).

## Supplementary Material

Supplementary Information
