# Supplementary material for: CRISPR/Cas9 cleavage of viral DNA efficiently suppresses hepatitis B virus
Source: Sci Rep. 2015 Jun 2;5:10833. doi: 10.1038/srep10833 (PMC4649911; doi:10.1038/srep10833)
Supplement: Supplementary Information [file srep10833-s1.pdf]

CRISPR/Cas9 cleavage of viral DNA efficiently suppresses hepatitis B virus

Authors: Vyas Ramanan[1, †], Amir Shlomai[2,12 †], David B.T. Cox[1,6,9, †], Robert E. Schwartz[1,3,4], Eleftherios Michailidis[2], Ankit Bhatta[2], David A. Scott[6,11], Feng Zhang[1,6,10,11*], Charles M. Rice[2,*], Sangeeta N. Bhatia[1,3,5,6,7,8,*]

Affiliations:

[1]Department of Health Sciences and Technology, Massachusetts Institute of Technology, Cambridge, MA 02139, USA.

[2]Laboratory of Virology and Infectious Disease, Center for the Study of Hepatitis C, The Rockefeller University, New York, NY 10065, USA.

[3]Department of Medicine, Brigham and Women's Hospital, Boston, MA 02115, USA.

[4]Division of Gastroenterology and Hepatology, Weill Cornell Medical College, New York, NY 10065, USA.

[5]Department of Electrical Engineering and Computer Science, Massachusetts Institute of Technology, Cambridge, MA 02139, USA.

[6]Broad Institute, Cambridge, MA 02139, USA.

[7]Howard Hughes Medical Institute, Cambridge, MA 02139, USA.

[8]Koch Institute for Integrative Cancer Research, Massachusetts Institute of Technology, Cambridge, MA 02139, USA.

[9]Department of Biology, Massachusetts Institute of Technology, Cambridge, MA 02139

[10]Department of Brain and Cognitive Sciences, Massachusetts Institute of Technology, Cambridge, MA 02139

[11]McGovern Institute for Brain Research, Massachusetts Institute of Technology, Cambridge, MA 02139

[12]Current address: The Liver Institute, Rabin Medical Center, Beilinson hospital and the Sackler Faculty of Medicine, Tel-Aviv University, Tel-Aviv, Israel

†These authors contributed equally to the work

Supplementary Materials

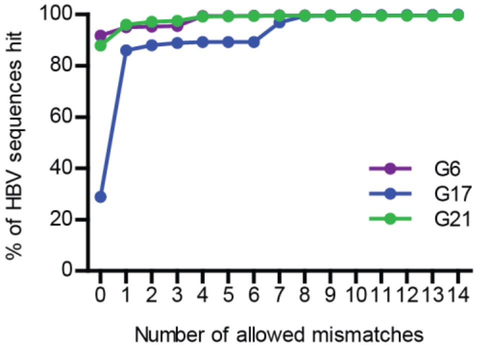

Figure S1: Guide RNAs targeting conserved regions target large majority of patient-derived virus genomes. All whole-genome sequences from HBV isolates were queried from GenBank to determine the conservation of 23 nt target sequence (20 nt spacer + 3 nt PAM) for 3 guides (6, 17, and 21). x-axis denotes number of allowed mismatches, and y-axis denotes the percentage of sequenced isolates that fall within this number of mismatches to native sgRNA target site.

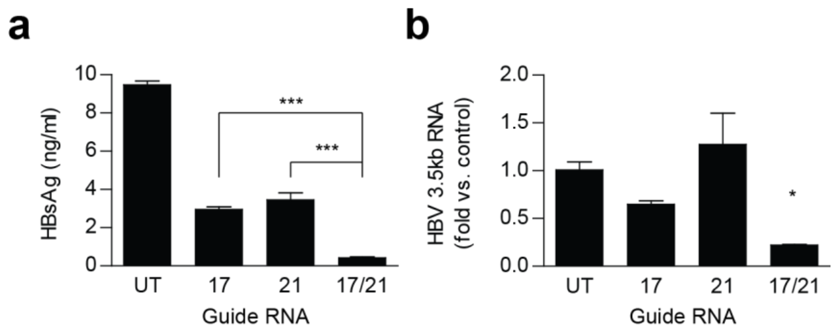

Figure S2: Multiplex targeting of HBV improves antiviral potency of CRISPR/Cas9. HepG2 cells were co-transfected with 1.3x WT HBV and sgRNA/Cas9-2A-mCherry construct, where the sgRNA is an untargeted control (UT), sg17, sg21, or a combination of sg17 and sg21. The combination of sg17 and sg21 reduces (a) HBsAg production and (b) HBV 3.5kb RNA relative to single targeting with sg17 and sg21. *$p < 0.05$ vs. all other groups, ***$p < 0.001$ vs. indicated groups.

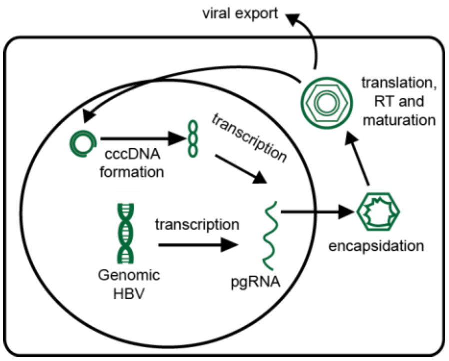

Figure S3: The HBV life cycle within HepG2.2.15 cells. HepG2.2.15 cells contain genomically integrated linear 1.3x WT HBV sequences, from which viral proteins and cccDNA are constitutively produced via transcription followed by translation (proteins) or reverse transcription and nuclear re-import (cccDNA). The persistent HBV production in this system

enables us to assay the long-term anti-HBV effects of CRISPR/Cas systems targeting viral DNA.

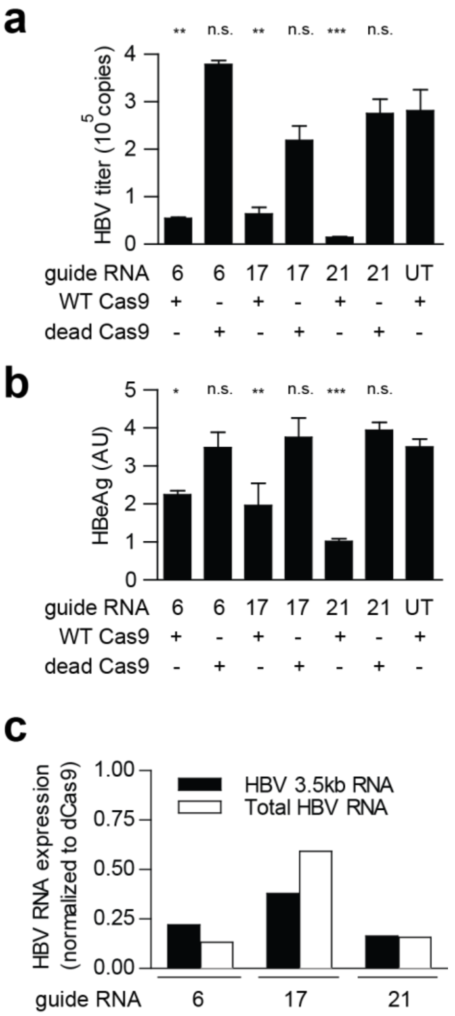

Figure S4: HBV products are reduced upon long-term CRISPR/Cas expression. Stable lines of HepG2.2.15 cells expressing 3 different on-target guides with nuclease-active or nuclease-dead Cas9, along with 3 non-HBV targeting guides, were seeded at consistent cell densities (20,000 cells/cm2) and allowed to secrete virions and viral proteins into the supernatant. 72h later, supernatant was collected and (a) viral titer and (b) HBeAg (a secreted protein produced from the C ORF, used clinically as a marker of active viral replication) were quantified. UT stands for untargeted guides, where scrambled sgRNAs were used instead of those targeting HBV. (c) Total HBV RNA and 3.5kb RNA (consisting of pregenomic RNA and the longest

translated HBV RNA species, which are difficult to distinguish) were quantified at 36 days post transduction, with HBV RNA suppression continuing out to this late time point. (a-b) *$p < 0.05$ vs. UT; **$p < 0.01$ vs. UT; ***$p < 0.001$ vs. UT as assessed by one-way ANOVA followed by Dunnett's post-hoc test.

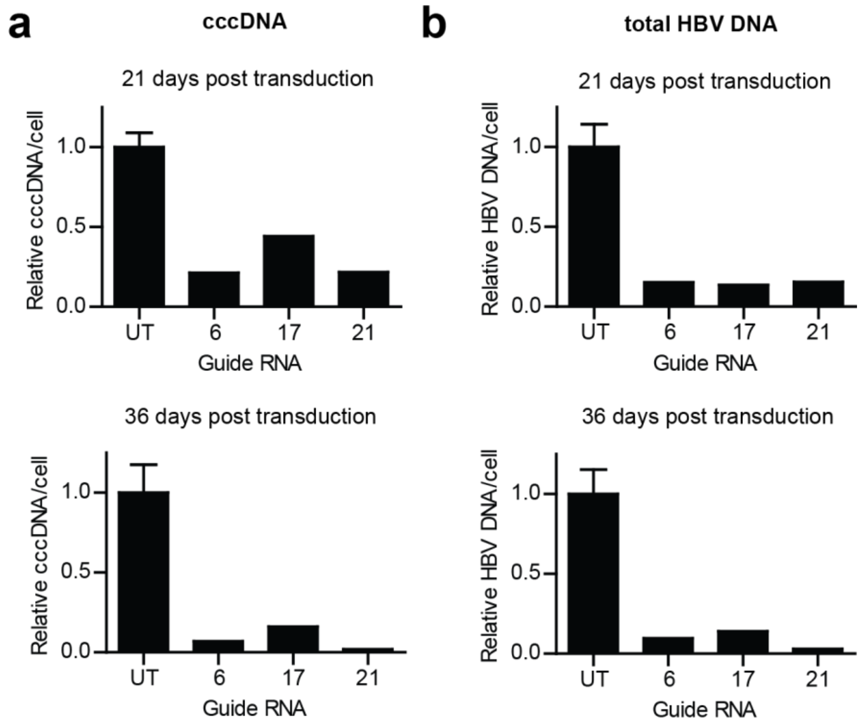

Figure S5: HBV DNA and cccDNA reductions upon long-term CRISPR/Cas expression are produced with multiple guides. (a) cccDNA reductions at 21 and 36 days post transduction across 3 guides (6, 17, and 21); large reductions are seen in each. (b) Total HBV DNA reductions at 21 and 36 days post transduction are also large across these 3 guide RNAs.

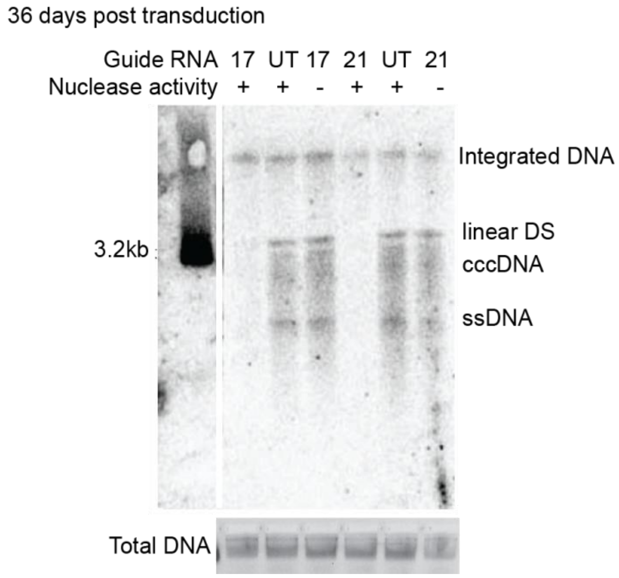

Figure S6: Southern blot analysis confirms that CRISPR-mediated reduction in viral DNA is specific for nonintegrated forms. At 36 days post transduction, DNA was isolated from HepG2.2.15 cells transduced with guide 17 or 21 or appropriate controls and a Southern blot was performed to detect HBV DNA. Comparing lanes 1 and 4 to the control lanes, it is clear that cccDNA and free linear double- and single-stranded HBV DNA are dramatically decreased, while the chromosomally integrated HBV DNA remains intact.

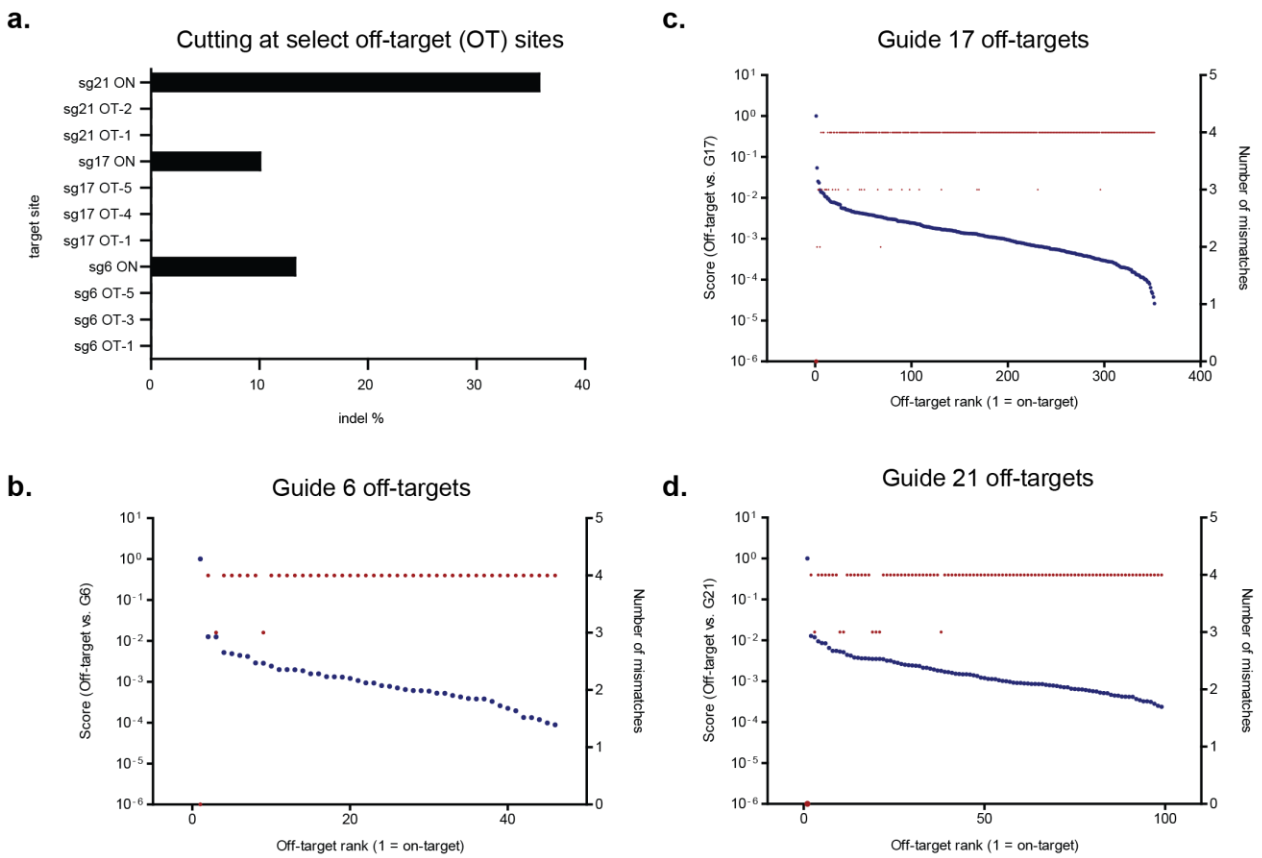

Figure S7: Lack of off-target effects at predicted potential off-target sites. (a) Viral and human genomic sequences around computationally predicted on-target and off-target sites were amplified from HepG2.2.15 cells that were lentivirally transduced with CRISPR/Cas9 constructs at over 4 weeks post transduction. Amplicons were deep sequenced to determine indel generation at off-target sites, and no indels were captured in any of the 8 off-target sites measured (with on-target indel fraction between 10-36%. (b-d) Computationally predicted off-targets for guides sg6, sg17, and sg21 with off-target rank on x axis and computationally determined off-target score (blue) and number of mismatches (red) on y axis.

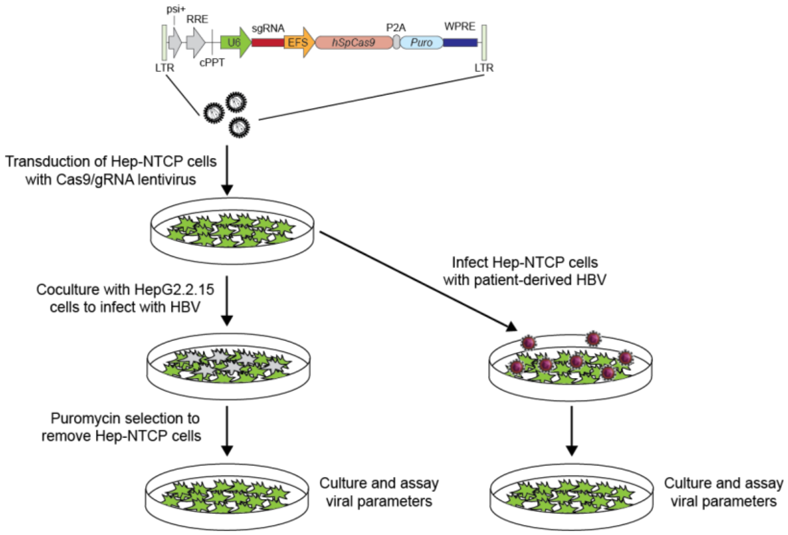

Figure S8: Schematics for *de novo* infection experiments. Hep-NTCP cells were transduced with Cas9/gRNA constructs containing either g17 or g17M (mutant of g17, resulting in 5bp DNA bulge upon complexation to HBV DNA target), and either WT or dead Cas9, and then selected with puromycin to generate stable lines. (Left) These cells were seeded in coculture with HepG2.2.15 cells, which produce infectious HBV virions that then infect the transduced Hep-NTCP cells. After transient coculture, HepG2.2.15 cells were killed by puromycin selection, and Hep-NTCP cells were cultured for several days and then harvested to assay viral parameters. (Right) These cells were infected with HBV virions derived from HBV+ patient plasma, then cultured and harvested to assay viral parameters.

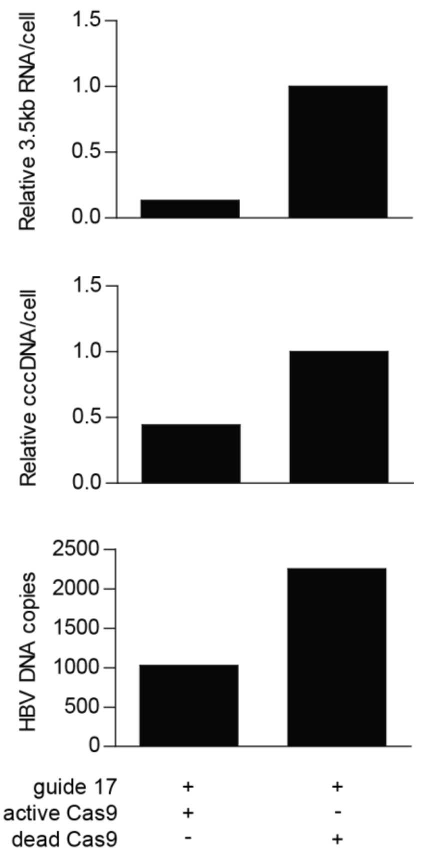

Figure S9: CRISPR/Cas-mediated disruption of HBV in patient-derived virus model system. Hep-NTCP cells (See Methods) were infected with HBV from infected patient serum upon transduction of guide 17 and active or nuclease-dead Cas9. 9 days after infection, the cells were harvested and viral products were quantified. Nuclease-active Cas9 caused decreases in HBV 3.5kb RNA, cccDNA, and total DNA levels.

Table S1: Target sequences against HBV genome

sgRNA	Target sequence (20 nt)
1	GACTTCTCTCAATTTTCTAG
2	GTTGGTGAGTGATTGGAGGT
3	GGCATAGCAGCAGGATGAAG
4	GGCTTTCGGAAAATTCCTAT
5	GCTGCCAACTGGATCCTGCG
6	GGGGCGCACCTCTCTTTACG
7	GAAGCGAAGTGCACACGGTC

8	GCAGAGGTGAAAAAGTTGCA
9	GTTGATAGGATAGGGGCATT
10	GTCGCAGAAGATCTCAATCT
11	GCCTGCTAGGTTTTATCCAA
12	GGAACAAGATCTACAGCATG
13	GGCGAGGGAGTTCTTCTTCT
14	GACCTTCGTCTGCGAGGCGA
15	CCTCCAAGCTGTGCCTTGGG
16	ATCGACCCTTATAAAGAATT
17	TAAAGAATTTGGAGCTACTG
18	CCCGTCGGCGCTGAATCCTG
19	GGGTTGCGTCAGCAAACACT
20	TTTGCTGACGCAACCCCCAC
21	TCCTCTGCCGATCCATACTG
22	CCGCTTGTTTTGCTCGCAGC
23	AACCCCCACTGGCTGGGGCT
24	CCTGCTGCGAGCAAAACAAG

Table S2: Primers used for *Surveyor* Assay

Guide 6-F: TATCCATGGCTGCTAGGCTG

Guide 6-R: AGTCAGAAGGCAAAAACGAGAG

Guide 17-F1: TATCCATGGCTGCTAGGCTG

Guide 17-R1: AGGGGCATTTGGTGGTC

Guide 17-F2: AAATTGGTCTGCGCACCAGC

Guide 17-R2: AGGTCTCTAGATGCTGGATCTTCC

Guide 21-F1: GGTTATCCTGCGTTAATGCCC

Guide 21-R1: GTCCGCGTAAAGAGAGGTG

Guide 21-F2: TGAACCTTTACCCCGTTGCCC

Guide 21-R2: AGAGAGTCCCAAGCGACCCC
